# Prolonged and high dosage of tigecycline – successful treatment of spondylodiscitis caused by multidrug-resistant *Acinetobacter baumannii*: a case report

**DOI:** 10.1186/s13256-017-1357-5

**Published:** 2017-07-08

**Authors:** Olga Tsachouridou, Adamantini Georgiou, Sideris Nanoudis, Theofilos Chrysanthidis, Georgia Loli, Petros Morfesis, Pantelis Zebekakis, Symeon Metallidis

**Affiliations:** First Internal Medicine Department, AHEPA University General Hospital, 1, Stilponos Kiriakidi Str, 54636 Thessaloniki, Greece

**Keywords:** Spondylodiscitis, *Acinetobacter baumannii*, Multi-drug resistance, Tigecycline, Prolonged administration

## Abstract

**Background:**

The incidence of infectious spondylodiscitis has been increasing over the last few years. This reflects the expanding elderly and immunocompromised populations and the rising implementation of invasive spinal procedures. Infection may be inoculated into the disc space directly during invasive spinal procedures. Osteomyelitis caused by *Acinetobacter* species is rare and mainly caused by multidrug-resistant strains.

**Case presentation:**

We present the case of a 72-year-old Greek woman with postoperative spondylodiscitis caused by a multidrug-resistant *Acinetobacter baumannii* strain that was successfully treated, after she declined surgical treatment, with prolonged and high dosage of tigecycline. She received intravenously administered tigecycline 200 mg per day for 60 days and then 100 mg per day for a total of 102 days and was infection-free.

**Conclusions:**

We reviewed the literature on the role of *Acinetobacter baumannii* as a cause of osteomyelitis, emphasizing the difficulty of treatment and the potential role of tigecycline in conservative treatment of the infection. We believe that 102 days in total is the longest time that any patient has received tigecycline in the literature, thus our patient is a unique case of successful treatment of spondylodiscitis.

## Background

In recent years, the incidence of infectious spondylodiscitis has risen due to improvements in health care and prolonged life expectancy. This infection is associated with older age, immunocompromised status, and presence of comorbidities. Infection may be inoculated into the disc space directly during invasive spinal procedures, of which 0.1 to 4.0% are complicated by septic discitis. This accounts for 20 to 30% of all cases of spondylodiscitis [1]. *Staphylococcus aureus* is the most commonly isolated pathogen in discitis complicating invasive spinal procedures (17 to 33%), followed by coagulase-negative staphylococci (13 to 29%), Gram-negative bacilli including *Pseudomonas aeruginosa* and *Stenotrophomonas maltophilia* (9 to 27%), streptococci (2 to 6%), and anaerobes, such *as Propionibacterium acnes* and *Peptostreptococcus* [2]. Osteomyelitis caused by *Acinetobacter* species is rare. A large number of cases have been reported in soldiers from Iraq. In most cases in the literature, infections were caused by multidrug-resistant (MDR) strains [3].

In the present report, a woman with postoperative spondylodiscitis caused by MDR *Acinetobacter baumannii* was treated successfully with prolonged and high dosage of tigecycline. She received tigecycline 200 mg per day for 60 days and then 100 mg per day for a total of 102 days.

## Case presentation

**Fig. 1:**
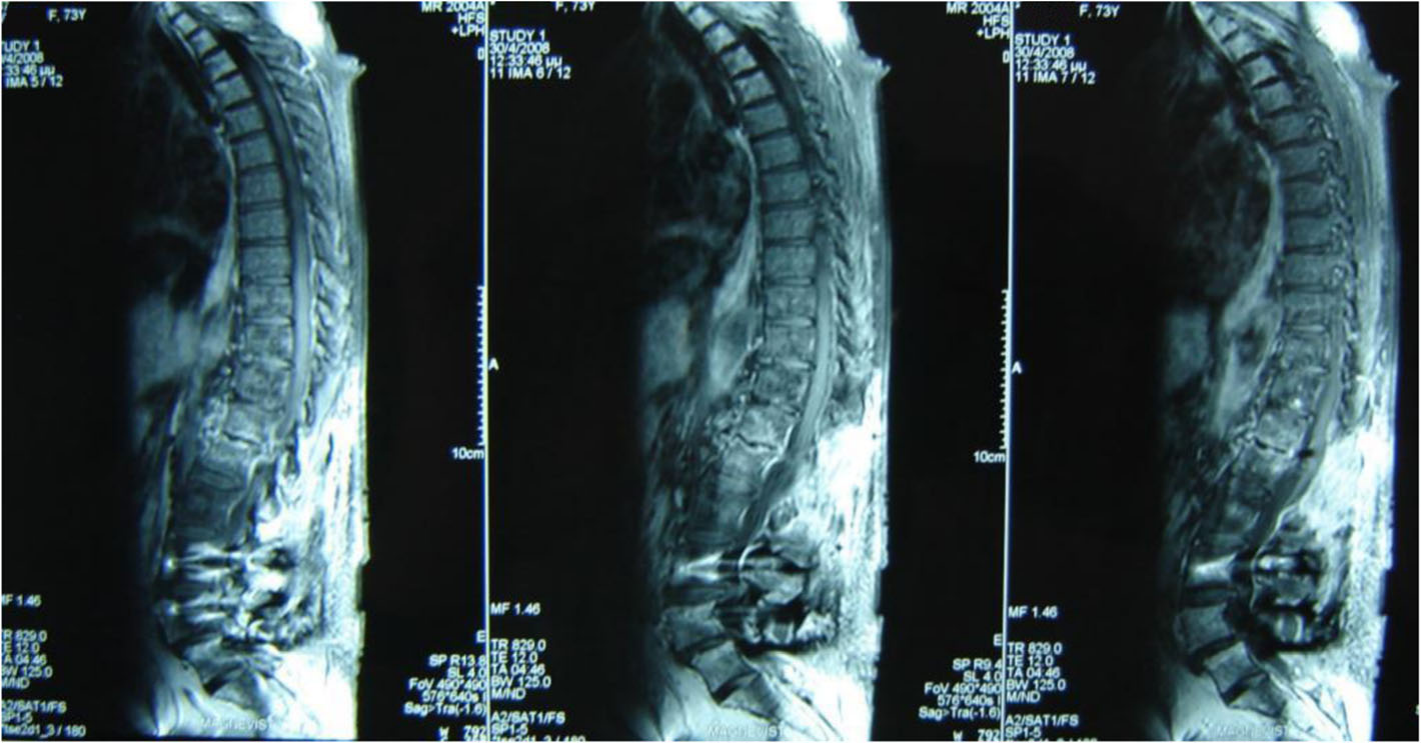
Magnetic resonance imaging showing signs of osteomyelitis at T12 and L1 vertebrae

A 72-year-old Greek woman with a medical history of serious allergic reaction to penicillin, chronic back pain, and hypertension well controlled on diuretic therapy, underwent a surgical operation for lumbar spinal stenosis, due to spondylolisthesis in lumbar vertebra 5 (L5), using instrumentation. She is a retired public employee and lives with her husband in a city in Northern Greece. She had not recently traveled outside Greece and she had no recent prior injuries. She did not drink alcohol, smoke tobacco, or use illicit drugs. On arrival, her arterial pressure was 135/80 mmHg and her other vital signs were normal. Her lungs were clear, and her heart sounds had a regular rhythm and were normal. Bowel sounds were present, and her abdomen was soft and tender on palpation. There was no rash or edema. Urine analysis was performed on admission and results were within the normal range. A neurological examination was performed with no abnormal findings: examination of cranial nerves; motor, sensory, reflex, and coordination assessment; examination of gait and station; and examination of mental status. During the operation, a discectomy was performed for a symptomatic disc herniation at thoracic vertebra 12–lumbar vertebra 1 (T12–L1). She did not receive prophylactic antibiotic treatment prior to surgery. Despite normal postoperative recovery, she was febrile on the second day (temperature up to 38.5 °C) with no clinical site of infection. Her white blood cells (WBC) count and C-reactive protein (CRP) were elevated: WBC count 14.61 cells/μL, CRP 10.2 mg/dL with normal reference range of 0.0 to 0.8 mg/dL. Remaining laboratory values were as follows: hemoglobin (Hb) 11.5 g/dL, platelets 165,000/mm^3^, blood glucose 98 mg/dL, serum sodium (Na) 142 mEq/L, serum potassium (K) 4.3 mEq/L, serum creatinine 0.9 mg/dL, total bilirubin 0.9 mg/dL, serum glutamic oxaloacetic transaminase (SGOT) 30 U/L, and serum glutamic pyruvic transaminase (SGPT) 26 U/L. Empirical therapy with levofloxacin was initiated; it was preferred due to her drug-allergy history. Blood and urine cultures were negative. A chest X-ray (CXR) was normal. She was afebrile by the fifth postoperative day and laboratory tests were normalized. She was discharged from hospital and continued levofloxacin 500 mg orally twice daily for 7 more days. She returned 30 days later with low grade fever (maximum temperature of 37.9 °C) and severe back pain that needed opiate analgesics. Her CRP was re-elevated (10.3 mg/dL). A magnetic resonance imaging (MRI) of her lumbar spine was performed that revealed facet joint surfaces of T12 and L1 vertebrae abnormalities, hypointense signal at T1-weighted images, and hyperintense signal in inversion recovery (IR) images. The T12–L1 disc showed hyperintense T2-weighted images signal. Intravenous contrast agent administration, revealed pathological signal at the facet joints and intervertebral disc characterized as inflammatory response. At the subcutaneous and soft tissue around the surgical intervention field, multicystic fluid areas could be observed. Post-intravenous paramagnetic contrast substance, ring formation around the fluid cysts and inflammatory response (signal enhancement) of the epidural soft tissue could be observed. No abnormal signal from the rest of the vertebrae was reported (Fig. 1). A bone biopsy was performed under fluoroscopy guidance. *A. baumannii* was isolated from all five cultures obtained (bone and soft tissue derived). *A. baumannii* was susceptible merely to gentamicin with a minimum inhibitory concentration (MIC) <2, to imipenem with a MIC of 1, and colistin with a MIC <4, and resistant to all other antibiotic agents tested.

Tigecycline (MIC 0.75) susceptibility was performed by Etest (AB Biodisk; Solna, Sweden); breakpoints were inferred from the available literature for *Enterobacteriaceae* (<2.0 is susceptible) as no current Clinical and Laboratory Standards Institute breakpoints are established. Despite our patient’s allergy history, she was originally administered imipenem intravenously, but she developed high fever, rash, and respiratory discomfort which were treated as an allergic reaction with H-1 histamine blockers and corticosteroids. Subsequently, tigecycline (50 mg twice a day, after loading dose of 100 mg) replaced imipenem and gentamicin (1 mg/kg administered intravenously three times a day) was added. Five days later, she developed severe vertigo and we decided to withdraw gentamicin.

She could not walk or do any other physical activity due to severe pain, but she refused any kind of surgical intervention that was suggested to her. Because of the lack of available data on the role of tigecycline in the treatment of osteomyelitis, especially for an infection caused by *A. baumannii,* we decided to double the dose of tigecycline (100 mg twice daily) after notifying our patient of the potential risks of higher doses of tigecycline (increased probability of developing tigecycline’s side effects such as nausea, vomiting, diarrhea, abdominal pain, pruritus, rash, headache, hepatotoxicity). She consented prior to starting enhanced dosage of tigecycline.

She had no adverse reactions and tolerated the regimen well, apart from slight nausea the first 2 days, which was managed with metoclopramide 10 mg administered intravenously. She was afebrile after 15 days and 30 days later she requested less opiate analgesics. Her CRP and erythrocyte sedimentation rate (ESR) were still elevated: CRP 5.7 mg/dL and ESR at 70 mm/hour. A new MRI, 30 days post the initiation of treatment with tigecycline revealed partial improvement in the soft tissue. She still refused any surgical intervention. She continued conservative therapy with high dosage for another 30 days, but then she developed severe hypoalbuminemia (serum albumin measuring at 1.9 g/dL while baseline serum albumin was 3.8 g/dL) and peripheral edema that resolved after reducing the dose to 50 mg twice daily. After completing 75 days of therapy, she could walk again and was free of analgesics, although her CRP and ESR were not yet normal. She was discharged from our hospital and continued tigecycline administered intravenously at home for a total 102 days. She had no infection relapse (clinical or radiographic signs) 18 months after the end of therapy and her CRP and ESR levels were finally normalized. Her remaining laboratory values were as follows: Hb 12.1 g/dL, WBC 5.4 cells/μL, platelets 283,000/mm^3^, blood glucose 88 mg/dL, serum Na 145 mEq/L, serum K 4.1 mEq/L, serum creatinine 0.8 mg/dL, total bilirubin 0.7 mg/dL, SGOT 35 U/L, and SGPT 29 U/L.

## Discussion

*A. baumannii* is a rare cause of osteomyelitis and especially of spondylodiscitis. Clinical management of *A. baumannii* bone infections in humans has not been well established, especially for MDR isolates. Only one case series study, by Davis *et al*., has been published regarding MDR *Acinetobacter* extremity infections in soldiers from Iraq [3]. All 18 patients with osteomyelitis underwent multiple surgical debridements of necrotic bone. Ten of the patients with osteomyelitis were treated with dual antimicrobial agents, seven with monotherapy, and one with surgical debridement alone. The primary combination of antimicrobial agents was imipenem (500 mg every 6 hours) in combination with high-dose amikacin (15 to 20 mg/kg daily). In a few instances, when imipenem was not active against the isolated organism, ampicillin/sulbactam or ceftazidime was used. They reported successful therapy in all cases with no relapses [3]. Schafer and Mangino published a case report of probable osteomyelitis caused by MDR *A. baumannii* treated successfully with tigecycline for 43 days. Again this patient underwent surgical debridement [4]. Sipahi *et al*. reported a case of postoperative spondylodiscitis due to MDR *A. baumannii* [5]. This patient received intravenously administered tigecycline for a total of 45 days, and was discharged with orally administered doxycycline, netilmicin, and sulbactam.

Besides the lack of clinical data, we had to address the refusal of our patient to undergo surgical debridement and the fact that tigecycline was the only therapeutic option due to allergic and adverse reactions to other antibiotic options. Furthermore, data regarding the bone concentrations of tigecycline are conflicting. Tigecycline concentration in bone has been evaluated in an experimental rat model and a single-dose human study [6]. The rat model showed an area under the curve (AUC) in bone ≈ 250× higher than plasma. The investigation in humans showed an AUC 0 to 24 ratio (site:serum) of 0.41/0.28 and the discrepancy was attributed to either tight binding of tigecycline to bone or poor extraction methods [7]. Bhattacharya *et al*. evaluated tigecycline bone concentrations in patients undergoing elective orthopedic surgery, using multiple doses [8]. The bone to serum ratio calculated using the AUCt values was 4.77, confirming tigecycline penetration into bone. The mean of all the bone concentrations reported in this study (range, 259 to 2262 ng/g) was 898 ng/g. The bone AUCt value was 11,465 ng h/g [8].

In addition, pharmacodynamic data suggest that an AUC/MIC >6.96 is more likely to lead to successful clinical and microbiological outcomes in patients with complicated intra-abdominal infections [9]. Koomanachai *et al*. in a pharmacodynamic evaluation of tigecycline against *A. baumannii* in a murine pneumonia model suggested that tigecycline doses of up to 200 mg/day may be required to provide adequate exposure for *A. baumannii* [10]. Based on these data we decided to administer high doses of tigecycline (100 mg twice a day) for 60 days. After 60 days of therapy, we were forced to reduce the dose due to severe hypoalbuminemia and peripheral edema. The symptoms resolved with reduction of dose to normal and our patient could continue the regimen for another 42 days. In a study by Griffin *et al*. the use of tigecycline was evaluated in 13 patients with osteomyelitis but none with *Acinetobacter* species. Eleven patients (85%) achieved clinical success. The median length of treatment was 6 weeks. Of the patients, 47% experienced some adverse event with the most prevalent being gastrointestinal in nature and five patients required discontinuation of treatment [11]. In another study by De Pascale *et al*., a high dose of tigecycline was evaluated in critically ill patients with severe infections due to MDR bacteria [12]. A high dose of tigecycline was given to 33 patients, 15 of them due to extensively drug-resistant (XDR) *A. baumannii*. There was no statistical difference for adverse events between standard and high dose of tigecycline. The clinical cure rate and microbiological eradication percentage were higher when tigecycline was used at higher doses (57.5 versus 33.3, *P* = 0.05; and 57.1% versus 30.4%, *P* = 0.07, respectively) [12].

## Conclusions

To the best of our knowledge, 102 days in total is the longest time that any patient has received tigecycline in the literature for any reason. Our patient is free of disease 18 months after the end of treatment despite multiple complications during her medical management. Further clinical and pharmacokinetic studies are required to assess and define the role of tigecycline in the treatment of osteomyelitis.
